# Giant Cell Arteritis Presenting as Unilateral Arteritic Anterior Ischemic Optic Neuropathy

**DOI:** 10.7759/cureus.16653

**Published:** 2021-07-27

**Authors:** Rahaf A Mandura

**Affiliations:** 1 Ophthalmology, King Abdul-Aziz University, Jeddah, SAU

**Keywords:** giant cell arteritis, vasculitis, loss of vision, arteritic anterior ischemic optic neuropathy, unilateral, visual loss

## Abstract

Giant cell arteritis (GCA) is a rare inflammatory vasculitis of unknown cause that involves large and medium arteries. Arteritic anterior ischemic optic neuropathy (AAION) is attributed to vascular occlusion of the posterior ciliary arteries (PCAs) which supply the optic nerve head (ONH). AAION is the most common ophthalmic complication of GCA and can cause sudden and irreversible loss of vision with a high risk of involvement of the second eye. A 57-year-old female patient presented with unilateral sudden onset visual loss in the right eye (OD) for two days. It was accompanied by severe right-sided headache and scalp tenderness on the right temple, neck as well as the presence of jaw pain over the past three months. Visual acuity (VA) was hand motion (HM) OD, and 20/20 in the left eye (OS). Fundus examination revealed diffuse swollen optic disc with pallid "chalky white" appearance OD and normal healthy optic disc OS. A dramatically elevated erythrocyte sedimentation rate (ESR) and C-reactive protein (CRP) were found. Therefore, a diagnosis of GCA was made, and immediate IV methylprednisolone was started followed by oral prednisone doses. A right temporal artery (TA) biopsy was done later and was negative. On follow-up, VA has maintained at HM level OD, and no involvement of the second eye occurred. GCA is a rare form of vasculitis that can be difficult to diagnose especially in the setting of negative TA biopsy. We support the evidence that negative TA biopsy does not rule out clinically suspected GCA with elevated ESR and CRP and recommend keeping a low index of suspicion as immediate treatment is required to prevent irreversible vision loss.

## Introduction

Giant cell arteritis (GCA) is a rare inflammatory vasculitis of unknown origin. It can involve the medium and large arteries associated with non-specific symptoms such as depression, fatigue, and weight loss. The most commonly affected blood vessels are the internal maxillary artery, superficial temporal artery (TA), and the orbital (retrobulbar) arteries which include the ophthalmic artery (OA), central retinal artery (CRA), and the posterior ciliary arteries (PCAs) [1]. Arteritic anterior ischemic optic neuropathy (AAION) is attributed to vascular occlusion of PCAs which supplies the optic nerve head (ONH). It is the most common ophthalmic complication of GCA and can cause sudden and irreversible visual loss. The vision is usually light perception or lower with third of cases being bilateral after one week [2].

The most significant risk factor for GCA is old age as it is rarely seen in patients younger than 50 years. In those 50 years and older, the incidence increases peaking at the eighth decade [1]. It represents a real ophthalmic emergency as the risk of visual loss is very high if not diagnosed and treated promptly with high-dose corticosteroid therapy. In addition, there is a risk of contralateral involvement. It has been reported that 65% of untreated AAION caused by GCA have developed involvement of the other eye within 10 days [3]. We report a case of unilateral AAION related to GCA in a woman in her fifth decade of life.

## Case presentation

A 57-year-old female patient complained of unilateral, sudden onset vision loss in the right eye (OD) for two days, accompanied by severe right-sided headache and scalp tenderness on the right temple and neck associated with jaw pain, especially while chewing, over the past three months. In addition, the patient reported myalgia, particularly in the upper legs and arms, and general fatigue. There were no other associated constitutional or systemic symptoms. Past medical history was significant for type 2 diabetes mellitus, hypertension, dyslipidemia, and hypothyroidism on medical treatment. The neuro-ophthalmological examination revealed that she was alert and oriented with normal vital signs. The best corrected visual acuity (VA) was hand motion (HM) in OD, and 20/20 in the left eye (OS). Pupil examination showed grade 3 relative afferent pupillary defect OD, and normal OS. Intraocular pressure was measured by air-puff tonometer and showed 16 mmHg OD, and 15 mmHg OS. Slit lamp examination of the anterior segments was unremarkable in both eyes. Dilated fundus examination revealed diffuse swollen optic disc with pallid "chalky white" appearance OD and normal healthy optic disc OS. Scattered familial drusen in the macular area were noted. Retinal vessels and peripheries were unremarkable in both eyes. The extraocular motility was full in both eyes. The right superficial TA was palpable with tenderness while Humphrey's visual field test showed total absolute scotoma OD, and normal OS.

The routine laboratory tests showed slightly decreased red blood cells of 3.67 × M/microL (normal 4-5.5 × M/microL) and hemoglobin of 10.7 g/L (normal: 12-15 g/L) and normal platelet count of 285 × 109/L (normal: 150-450 × 109/L) while there was an elevation in erythrocyte sedimentation rate (ESR) of 65 mm/h (normal: 0-15 mm/h) and C-reactive protein (CRP) of 20.1 mg/L (normal: 0-3 mg/L).

**Figure 1:**
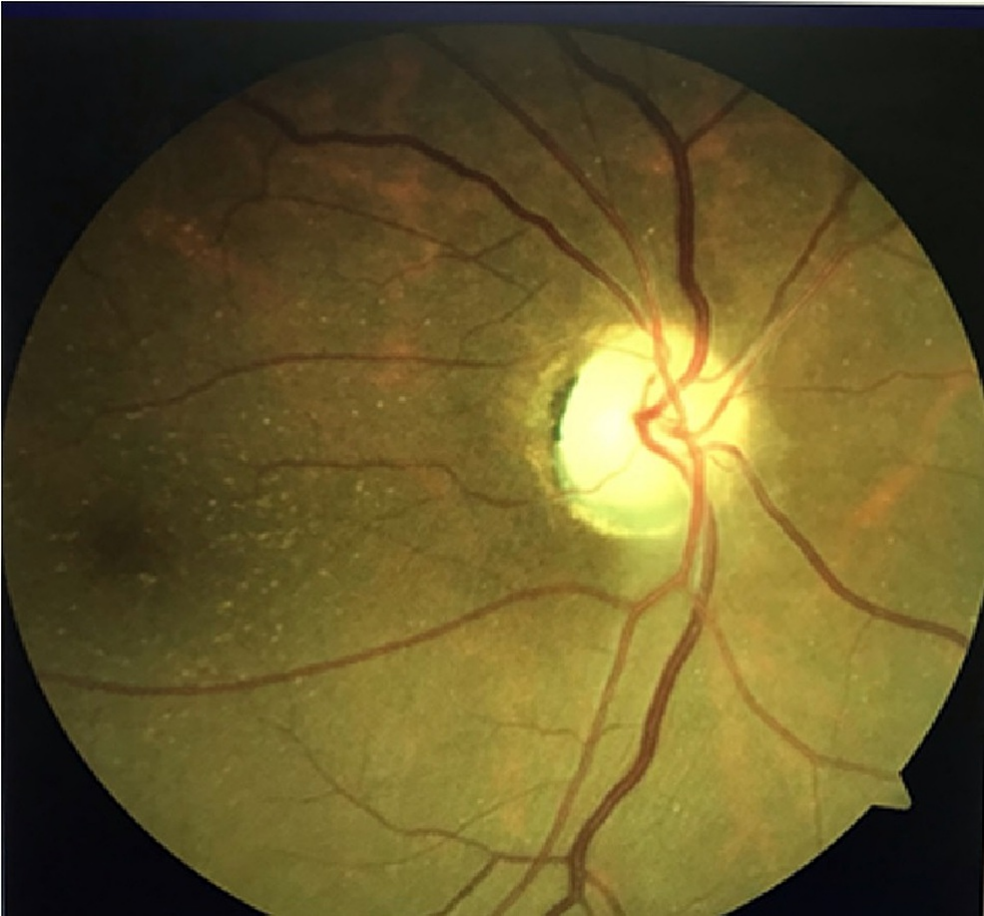
Fundus photo of the right eye showing chalky white optic nerve head pallor representing the sequelae AAION. AAION, arteritic anterior ischemic optic neuropathy

**Figure 2:**
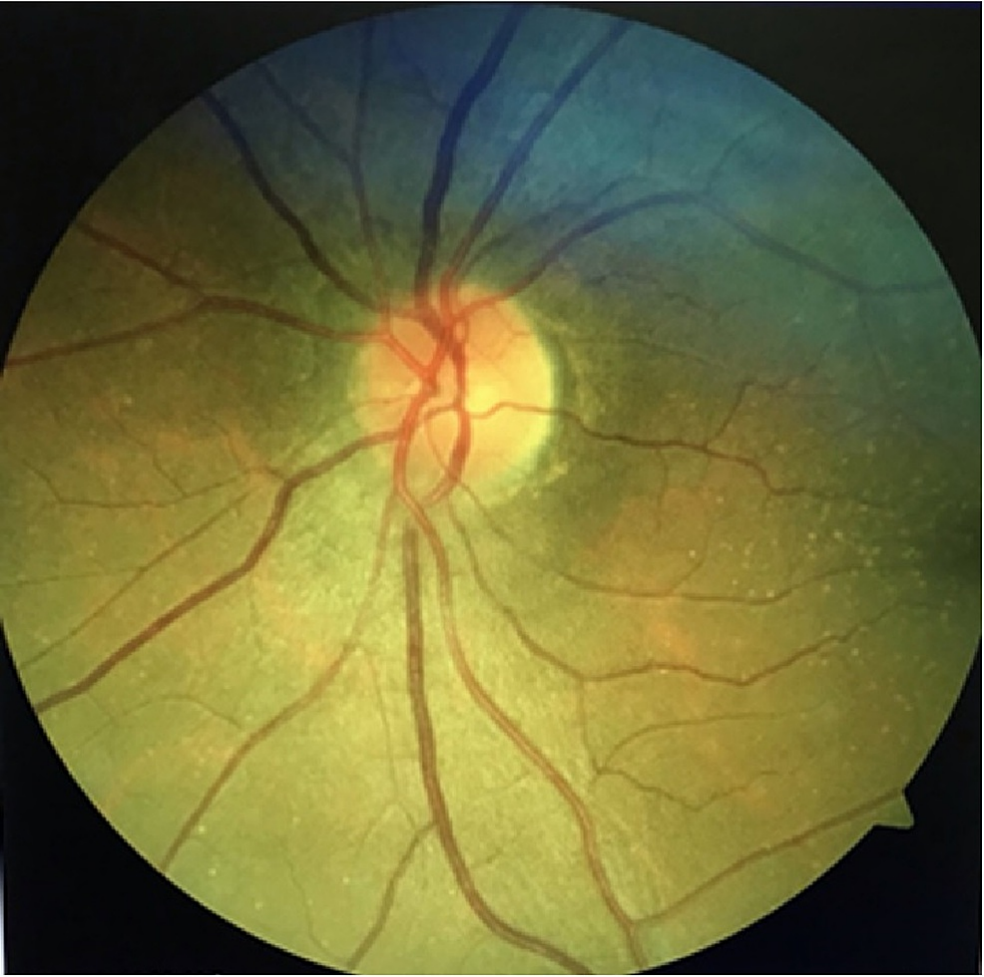
Fundus photo of the left eye showing normal healthy optic nerve head.

The clinical and laboratory findings had raised our suspicion for GCA. Therefore, we started the patient immediately on IV methylprednisolone 1 g/day for three days followed by oral prednisone 1 mg/kg/day. The right temporal artery biopsy (TAB) was arranged and the earliest availability to be performed was three weeks after the initiation of the treatment. Later on, the histopathological examination came back negative. In addition, MRI of the brain and orbit was unremarkable for inflammatory and space-occupying lesions. The VA was maintained on HM level OD after treatment, the headache and jaw pain disappeared rapidly, and ESR and CRP went back to a normal level. The oral prednisone tapered slowly over a year till we reached a dose of 7.5 mg/day. Keeping the patient on maintenance low dose prednisone was recommended as she had a re-elevation of ESR level when oral prednisolone was stopped completely. In addition, methotrexate 15 mg/day was added as an immunosuppressive agent for long-term treatment. At two years follow up, VA was stable at HM OD and 20/20 OS, and fundus examination OD showed the sequelae of AAION with diffuse chalky white optic disc pallor OD (Figure 1), while OS showed normal healthy unaffected optic disc (Figure 2).

## Discussion

Giant cell arteritis is a rare disease in the Middle East and the Saudi population [4]. There is no available data estimating the incidence of GCA in Saudi Arabia due to the lack of studies as there has been no nationwide population-based study performed before. However, Bosley and Riley have reported only four positive TABs at a tertiary center in Saudi Arabia in 15 years period compared to a significantly larger number of positive TABs which was reported from similar North American studies in a much smaller population size communities [4]. The Bascom Palmer Eye Institute reported 185 positive biopsies over a 13-year period [3], while the Ochsner Clinic had 97 positive biopsies over a period of 19 years [5].

Segmental ischemia is represented by AIONs of the ONH which is supplied by the PCAs [6-7]. AIONs can be categorized into arteritic-AION and non-arteritic-AION [6-7]. Clinically, arteritic-AION is similar to non-arteritic-AION with few minor differences [6]. Visual loss is often found more severe in arteritic-AION than in non-arteritic-AION. In one study, 54% of patients with arteritic-AION related to GCA had variable VA that ranged from counting fingers to no light perception. On the other hand, 26% in the non-arteritic AION group had VA of only light (29%) or no light perception (4%) [6]. This result proves that sudden, painless, severe visual loss is remarkably suggestive of arteritic-AION [6-7]. This is going in concordance with our patient who has a poor presenting VA of HM. Also, systemic symptoms of GCA may precede visual loss by weeks or months similar to our patient’s case.

Referring to the criteria of the American College of Rheumatology [8], GCA should be suspected in all patients with arteritic-AION who are older than 50 years of age, with new headache in the temporal area, temporal arteries tenderness, and/or reduced pulse, jaw claudication, systemic symptoms (malaise, fever, etc.), ESR exceeding 50 mm/h, and typical histological findings (granulomatous involvement) in TAB. However, negative histological findings in TAB do not exclude the diagnosis of GCA [9] which was clearly demonstrated in a study conducted at Bowling et al. [9] who found that 78.4% of the clinically suspected GCA patients who performed TAB within one week of the onset of symptoms had a negative test, 15.7% of them had a positive test, and six patients had insufficient results. Furthermore, they found that 81.5% of the clinically suspected GCA patients who performed a TAB after one week of the onset of symptoms had a negative test, 3.7% of them had a positive test, and four patients have insufficient results [10].

The high levels of ESR and CRP, which are found in our patient’s case, are considered the most highly predictive laboratory data of GCA, with a combined sensitivity of up to 99.2% [10-11]. Normal values of these data in the context of low clinical suspicion are enough to safely rule out GCA and TAB is not necessary in these cases [10-11]. The visual prognosis is extremely unfavorable in acute unilateral arteritic-AION, and the visual outcome did not improve in the patients in other studies [12-14].

## Conclusions

Giant cell arteritis is a rare form of vasculitis that can be difficult to diagnose especially in the setting of negative TAB. We support the evidence that negative TAB does not rule out clinically suspected GCA with elevated inflammatory markers including ESR and CRP. Many studies in the literature have reported low biopsy positive rates and this concurs with our own result in this case. It is, therefore, important for clinicians to keep a low index of suspicion for this disease as immediate treatment is required to prevent irreversible vision loss and involvement of the second eye. 
